# Take it with a pinch of salt—ESG rating of stocks and stock indices^☆^

**DOI:** 10.1016/j.irfa.2022.102308

**Published:** 2022-10

**Authors:** Szilárd Erhart

**Affiliations:** Joint Research Centre of the European Commission, Competence Centre on Composite Indicators, Via E. Fermi, 2749, 21027 Ispra VA, Italy

**Keywords:** Sustainable finance, ESG rating, Stock index

## Abstract

This paper investigates the environmental, social, and governance (ESG) ratings of 20 leading stock exchange indices by analyzing and aggregating ratings of underlying stocks. ESG ratings are increasingly important inputs to sustainable investments in the European Union and United States with the phasing-in disclosure regulations. We find that ratings from two different rating providers (Sustainalytics and Refinitiv) for the same listed stocks are only weakly correlated, even if the scaling differences of the ratings are adjusted. Monte Carlo simulations are conducted to estimate how the choice of major ESG rating inputs (i) aggregation formula, (ii) weighting scheme and (iii) data provider influence the uncertainty of ratings and thus indirectly the sustainable investment process. The simulations reveal that the uncertainty is primarily related to choice of the ESG rating provider. We found that the popular best-in-class portfolio selection could be built on ESG scores. In lower segments of the ESG asset universe, investment selection becomes more challenging due to the increasing uncertainty of ratings. Finally, the paper shows that exchanges in the European Union provide relatively good ESG investment opportunities in international comparison.

## Highlights

• Low correlation of E, S and G scores is a barrier to ESG investments.
• Uncertainty of ESG ratings is related primarily to the choice of ESG rating provider.
• Best-in-class investment rules could be built on ESG scores of Sustainalytics and Refinitiv.
• Exchanges in the European Union provide good ESG investment opportunities in international comparison.

## 1 Introduction

Environmental, social, and governance (ESG) ratings have been becoming an integral part of financial, business and consumption decisions. Their importance has been acknowledged by new European and American political leaders recently. In Europe the European Commission’s president Ursula von der Leyen unveiled the European Green Deal in 2019 following the publication of the Union’s Sustainable Finance Strategy in 2018. In the US, the elected president Joe Biden has pledged USD2 trillion in climate spending in 2020.

Earlier research papers related to ESG ratings find an evident lack in the convergence of ESG measurement. These papers argue that investors and scholars should reopen the discussion about the concepts and practice of ESG scores to support the sustainable finance community reach their self-imposed objectives with the ESG measurement, Dorfleitner, Halbritter, and Nguyen (2015) and Drempetic, Klein, and Zwergel (2020).

Berg, Kölbel, and Rigobon (2019) show the importance of three factors in ratings: scope, measurement and weight and conclude that measurement divergence explains more than 50 percent of the overall divergence. Measurement divergence refers to the situation where rating agencies measure the same attribute using different indicators. For example, a firm’s labor practices could be evaluated on the basis of workforce turnover, or by the number of labor cases against the firm. Both capture aspects of the attribute labor practices, but they are likely to lead to different assessments.

yi Yu and Luu (2021) use the Bloomberg ESG disclosure score as the measure of transparency, and find that firm characteristics explain most of the variation in firms’ ESG disclosure, whereas variations in country factors such as corruption and political rights explain less.

In this study we investigate the sustainability characteristics of 20 leading global stock exchange benchmarks by analyzing and aggregating the Environmental Social Governance scores for the stocks in the benchmarks. Our key objective is to understand empirically the uncertainty of ESG ratings. We use data from two rating providers: Sustainalytics and Refinitiv. The key instrument in our empirical setup is the Monte Carlo simulation framework described in Section 2.1 on Methods. The simulations reveal that the uncertainty is primarily related to choice of the ESG rating provider. We discuss theoretical challenges of investments in a multivariate ESG assessment using indifference curves in Section 2.2. Furthermore, our study is related to research on composite indicators, (OECD-JRC, 2008). In particular, to those dealing with the design, uncertainty and sensitivity analysis of composite indicators and ratings, Saisana, Salitell, and Tarantola (2005), Becker (2021).

An important novelty of our study is that it extends the scope of the ESG discussion from ESG benchmarks to general benchmarks as these constitute the majority of the benchmark universe. MSCI and other financial benchmark developers have been active in designing ESG indices for investors and asset managers. Our study aims at creating a level playing field by treating traditional benchmarks in the same way as ESG benchmarks. Hence, it increases the transparency across the whole index universe as recommended by the European Commission (2019). The 20 global stock indices analyzed in our paper are becoming increasingly important with the growing popularity of passive investments strategies provided by Exchange-Traded Funds (ETFs) tied to these indices.

The rest of the paper is structured as follows. Section 2 describes the methods and the materials for the assessment of ESG ratings together with the theoretical and practical challenges of managing possible trade-offs between environmental, social and governance issues. Section 3 presents the empirical results by E, S and G components, by industries, and by stock indices. Furthermore, Section 3 also details our Monte Carlo simulation experiment to test the uncertainty of ESG scores. Section 4 summarizes the policy relevance and phasing-in regulations related to ESG ratings. Section 5 discusses the limitations of our empirical set-up and Section 6 concludes.

## 2 Materials and methods

### 2.1 Methods

The development of ESG ratings, like any measurement, entails assumptions and subjective decisions. Hence, one of the key objectives of our research is to test whether and to what extent some of the assumptions in the ESG assessment influence the ESG values of stock and stock indices, within a range of plausible alternatives in an uncertainty analysis similarly to Becker, Norlén, Dijkstra, and Athanasoglou (2020), Erhart, Becker, and Saisana (2019). We performed the Monte Carlo experiment and re-built an aggregated $ESG_{i}$ score for each stock 4000 times as defined in Eq. (1), where i denotes the stock, ENV, SOC and GOV denote the  *environmental, social and governance* scores, and *k* denotes the rating provider company. In each simulation run we randomly-selected combinations of three assumptions as detailed below.

(1) $$ESG_{i}=A\left\{w_{1}\cdot ENV_{i,k},w_{2}\cdot SOC_{i,k},w_{3}\cdot GOV_{i,k}\right\}$$

**Assumptions tested in the Monte Carlo simulations**

**Aggregation formula** The first assumption we varied was the aggregation formula denoted by the aggregation operator *‘A’* in Eq. (1). The aggregation operator was randomly varied and scores were either aggregated by the arithmetic or the geometric mean or the harmonic mean. In practice, rating providers aggregate the E, S and G scores into a single ESG score by using the weighted arithmetic mean. The geometric mean was chosen as an alternative approach, which is a non-compensatory aggregation method. In this way high scores in one component of the ESG rating does not compensate low scores in another, which is an alternative way to look at the ESG issue. For instance, if an issuer scores high on environmental indicators, it cannot offset its weak performance on social or governance ones (see further details of the aggregation rule in Section 2.2).
**Weights** The second assumption which was tested was the weighting scheme. Nominal weights assigned at the dis-aggregated level are all equal ($w_{1}=w_{2}=w_{3}$=1/3) in the Sustainalytics methodology and sector specific in the Refinitv ESG methodology. Therefore, the effect of randomly varying weights by ＋/−25% around the equal weights is tested, to investigate the effect of minor variations in the importance of different ESG components. To see what happens if some components are given zero weight, the variation scale for weights was allowed to be wider (＋/−100%) in a ceteris paribus separated experiment. Gaussian noise was added on the weights, while the total weight was constrained to add up to one [∑w = 1].
**Data provider** The third assumption we tested was the data provider. ESG investors have a free choice to select their preferred data provider. However, contrary to credit ratings, the precision and efficiency of ESG ratings cannot be judged on the basis of back-testing. As an analogy of credit ratings, there are no observations on outcome variables such as default events in case of credit ratings. To test the uncertainty faced by an uninformed investor from data provider selection, we varied randomly the data provider of the ESG scores between Sustainalytics and Refinitiv. As the scale and direction of scores in the Sustainalytics and Refinitv methodology are different, we made them comparable by changing the direction of the Sustainalytics risk scores and by normalizing these scores within industries (see detailed explanation about the conversion in Section 2.3 on Data).

Our robustness test of ESG ratings entailed an additional step, the sensitivity analysis. In modeling the sensitivity analysis is usually the last step following the uncertainty analysis. It estimates which of the input uncertainties are driving the output uncertainty, and by how much. Although uncertainty analysis may contain some information on the importance of assumptions, sensitivity analysis is still necessary to better understand the relative importance of assumptions as they interact with one another. Hence, one must vary uncertain parameters and assumptions simultaneously.

We applied variance-based sensitivity analysis which is largely considered as the “gold standard” in testing the effects of uncertainties in modeling and often missing from empirical papers, Becker (2021), Saltelli et al. (2019). In general, the central idea is that the uncertainty in a single output y of a model can be encapsulated as its variance V(y). As the variance increases, so does become the output more uncertain. Solob (2001) showed that variance in outputs can be decomposed into parts which are attributable to each uncertain input. Here, inputs can be considered as important assumptions of the rating model on aggregation, weighting, input data, etc.

(2) $$V\left(y\right)=\underset{m}{\sum }V_{m}+\underset{m}{\sum }\underset{l>m}{\sum }V_{m,l}+\cdots +V_{1,2,\ldots ,d},$$

where:

$$V_{m}=V\left[E\left(y|x_{m}\right)\right]$$

$$V_{m,l}=V\left[E\left(y|x_{m},x_{l}\right)\right]-V\left[E\left(y|x_{m}\right)\right]-V\left[E\left(y|x_{l}\right)\right]$$

and so on for the higher order terms. Here, V(⋅) denotes the variance operator, E(⋅) the expected value.

Saisana et al. (2005) suggested that one can apply variance based sensitivity analysis on composite indicators. ESG ratings are in practice composite indicators as they aggregate and weight indicators. From Eq. (2) one can derive the  *first order sensitivity index* $S_{m}$ in Eq. (3), which measures the fraction of the output variance caused by each uncertain input assumption alone. $S_{m}$ is defined as the unconditional output variance that is accounted for by the uncertainty in the underlying indicators ($x_{m}$).

(3) $$S_{m}=V_{m}/V\left(y\right),$$

Another variance-based measure is denoted $S_{Tm}$, and is called the *total order sensitivity index*.

(4) $$S_{Tm}=1-\frac{V\left[E\left(y|x_{-m}\right)\right]}{V\left(y\right)}=\frac{E\left[V\left(y|x_{-m}\right)\right]}{V\left(y\right)}$$

where $x_{-m}$ is the set of all inputs except $x_{m}$. The total order sensitivity index measures the contribution to V(y) of a given input $x_{m}$, as well as all its interactions of any order with other inputs.

Applying the sensitivity analysis method on ESG ratings requires substituting aggregated ESG scores into the output variable (y), calculate its variance V(ESG) and decompose this variance into the first order sensitivity index and the total order sensitivity index interactions.

### 2.2 Indifference curves of E, S and G scores

The two ESG rating providers in our sample (Sustainalytics, Refinitiv) apply additive aggregation formula to calculate the total ESG scores. Such a choice is common in the international practice of composite indicators, however, it has important consequences. An undesirable feature of additive aggregations is the implied full compensation, such that poor performance in some indicators can be compensated for by sufficiently high values in other indicators (OECD-JRC, 2008).

**Fig. 1:**
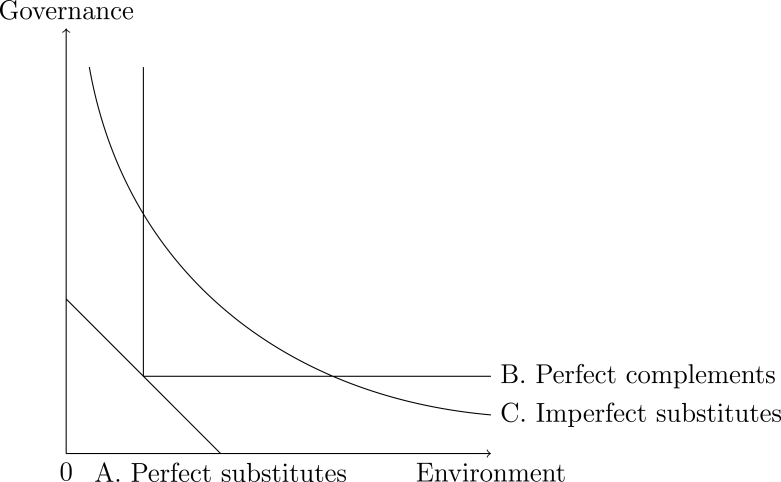
Example of an ESG indifference curve.

It is both a theoretical and practical challenge for citizens, regulators and investors, how they think about the indifference curves of environmental, social and governance issues (Fig. 1). Do they think environmental degradation can be perfectly substituted for example by social benefits in terms of less workplace injuries (curve A on Fig. 1)? Or just imperfectly substituted (curve C on Fig. 1)? Or are they complementary like oxygen and water for humans, we need both for living (curve B on Fig. 1). ESG ratings do not measure impacts directly on the environment or the social landscape. They measure the company’s economic value at risk driven by ESG factors (Sustainalytics) or the company’s relative ESG performance compared to industry peers (Refinitiv). Hence, they provide information indirectly on the possible implied environmental and social impacts, which may also govern the economic value at risk. Section 3.5 discusses the uncertainty of scores resulting from the aggregation rule choice (compensatory vs. non-compensatory) based on a Monte Carlo simulation exercise.

In general, the desired ideal or utopia option is often nonexistent. The ESG world is probably not an exception, e.g. for this multifaceted problem, there is no optimal solution for all criteria at the same time. Thus, best compromises have to be discovered.

### 2.3 Data

The two sources of our ESG rating dataset were Sustainalytics published by Yahoo Finance and Refinitiv. Sustainalytics is a global leader in ESG and Corporate Governance research and ratings. Refinitiv, formerly known as Thomson Reuters, is a London Stock Exchange Group business, a global provider of financial market data and infrastructure. Python program language was deployed to obtain data for the general stock index components and their ESG ratings for 20 exchanges in November 2020 and in April 2022 ( Table A.5 in Appendix). For the regression analysis in the paper the general company data was also sourced from Sustainalytics (book value, dividend yield, in %). Furthermore, country level sovereign credit default swap, (CDS, in basis points, bp) was collected from and the ESG disclosure rate (in %) of stock exchanges from Corporate Knights (Knights, 2020).

In the sample there are 1016 stocks which were rated by Sustainalytics in 2020 and 974 in 2022, and there are 722 stocks rated by Refinitiv in 2020 and 970 in 2022. The sample covers about 60 percent of the stocks in the analyzed indices, as currently no ESG rating is available for the remaining 40 percent of the publicly listed companies in the sample of the 20 stock indices ( Table 1, Appendix).

There are 15 European stock indices in the sample (Austria, Belgium, Denmark, Finland, France, Germany, Hungary, Italy, the Netherlands, Norway, Russia, Spain, Sweden, Switzerland, United Kingdom), 2 from North America (United States, Canada), Australia and 3 from Asia (Hong Kong, China and Japan).

The Sustainalytics ESG score and its subscores are absolute measures, meaning that a ‘high risk’ assessment reflects a comparable degree of unmanaged ESG risk across all covered subindustries, (Sustainalytics, 2019). Refinitiv produces sector specific ESG scores between 0 and 100, (Refinitiv, 2022). These scores are based on relative performance of ESG factors with the company’s sector (for environmental and social) and country of incorporation (for governance). Refinitiv does not presume to define what ‘good’ looks like; they let the data determine industry-based relative performance within the construct of their criteria and data model

It follows that there are three major differences between the ESG scores of Sustainalytics and Refinitiv which have to be addressed before the empirical analysis. The first difference is that Sustainalytics ESG scores are sustainability risk scores (the lower the score, the better) and Refinitiv’s ESG score scale direction is the other way around (the higher the score, the better). Hence, the Sustainalytics ESG risk scores are also converted into ESG scores by changing the direction of the measurement scales. The second important difference is that Sustainalytics calculates scores which are comparable across industries, while Refinitiv’s scores are sector specific. The third difference is that the scales of Sustainalytics scores is narrower for the subscores and the aggregated ESG scores as well. To correct the second and third differences, Sustainalytics scores were recalculated by normalizing scores for each stock with the min–max normalization method within the industry.

The data, Python web-scraping code and R script for the Monte Carlo simulations can be downloaded from a Mendeley repository dedicated to the research: [DOI: 10.17632/58mwkj5pf8.2,].

## 3 Results

### 3.1 Descriptive statistics

Table 1 shows the summary statistics for the original ESG scores with the converted Sustainalytics ESG scores. On average Sustainalytics environment risk scores (5.8) are somewhat lower than social risk scores (9.3) and governance risk scores (7.5) although the difference is not significant. The range of subcores is also somewhat wider for the environment score (0–31) than for the Social score (0–30.5) and the governance score (3–19).

The summary statistics of E, S and G subscores of Refinitiv are very similar (mean score of 66–68, standard deviation of 18–21 scores), although the mean scores of Refinitiv are somewhat higher than that of the Sustainalytics converted scores, which could be a result of non-overlapping observations in the Sustainalytics and Refinitiv samples.

**Table 1:** Summary statistics of Reifinitv and Sustainalytics ESG scores in the sample.

	Mean	Std. dev.	Min.	Max.	N(2020)	N(2022)
	Sustainalytics official absolute risk scores${}^{a}$					
Environmental	5.8	5.5	0	31.1	1016	974
Social	9.3	4.0	0	30.5	1016	974
Governance	7.5	2.7	0	18.6	1016	974
ESG	22.7	7.9	5	70.1	1016	974

	Sustainalytics reversed, industry specific scores${}^{b}$					
Environmental	60.8	26.3	0	100	1016	974
Social	55.6	24.5	0	100	1016	974
Governance	58.5	25.4	0	100	1016	974
ESG	56.9	23.9	0	100	1016	974

	Refinitiv official, industry specific scores					
Environmental	66.2	21.7	0	98	722	970
Social	67.7	19.9	0	98	722	970
Governance	68.9	18.3	1	98	722	970
ESG	66.3	17.7	4	100	722	970

**Notes:**${}^{a}$The upper block of the table reports the sample summary statistics of Sustainalytics official ESG risk scores. The lower the score the smaller the unmanaged ESG risk. This score is an absolute score and comparable across industries. ${}^{b}$The middle block shows converted Sustainalytics ESG scores, which were generated by changing the direction of the measurement scales and by calculating within industry group normalized scores for each company with the min–max normalization method. The bottom block shows ESG scores of Refinitiv, which is a sector specific normalized score comparable to the converted Sustainalytics scores in the middle block.

### 3.2 Correlation analysis

Earlier studies discussed correlation of ESG ratings of different providers. Berg et al. (2019), Gibson, Krueger, Riand, and Schmidt (2019) showed that there is no agreement between rating providers (0.4–0.6 pairwise correlation of ratings) which is much lower than correlations among credit ratings exceeding 0.9. Our correlation analysis confirms earlier results, as the pairwise correlation between ESG scores from Sustainalytics and Refinitiv is weak, in the range of 0.2–0.3, although the correlations are significant at standard significance levels (α = 1%).

Here, we focus more on the correlation structure of the ESG subscores (Table 2). In the ideal case, there should be positive significant correlations within the ESG aggregated score and subscores, (OECD-JRC, 2008). Both the Refinitiv and Sustainalytics subscores comply with the above requirement, as environmental, social and governance scores’ correlation ratios with the ESG score are balanced and vary within the recommended 0.4–0.8 range for meaningful aggregates. However, the pairwise correlation between Refinitiv and Sustainalytics scores on the E, S and G pillar level is weak (0.1–0.3). Furthermore, Sustainalytics environmental scores’ association with social and governance scores is not very strong, and this may limit opportunities in sustainable finance (see Table 2). . In a portfolio theory view, investors may target maximizing their return combined with some constraints on the aggregated ESG score. However, if the investor would like to do good on every ESG front and considers that the environment score, the social and government scores are not substitutes of each other but complements, the available investment universe becomes reduced (see further discussion in Section 2.2).

**Table 2:** Cross-correlation table.

Variables	R_E	R_S	R_G	R_ESG	SA_E	SA_S	SA_G	SA_ESG
R_E	1.000							
R_S	0.838	1.000						
R_G	0.429	0.451	1.000					
R_ESG	0.474	0.479	0.448	1.000				
SA_E	0.164	0.185	0.163	0.150	1.000			
SA_S	0.154	0.150	0.140	0.126	0.329	1.000		
SA_G	0.118	0.154	0.257	0.206	0.212	0.404	1.000	
SA_ESG	0.231	0.240	0.237	0.225	0.594	0.780	0.619	1.000

**Notes:** In the table above, ‘SA’ denotes Sustainalytics and ‘R’ denotes Refinitiv, ‘E’, ‘S’ and ‘G’ are for Environmental, Social and Governance. For ensuring comparability between Refinitiv and Sustainalytics scores, the Sustainalytics ESG absolute risk scores were transformed by changing the direction of the measurement scales and by calculating within industry group normalized scores for each company with the min–max normalization method. In total 928 observations were used for the calculations.

### 3.3 Sectoral ratings

Refinitiv publishes only industry specific ESG scores, hence this subsection details the analysis of Sustainalytics data. The Sustainalytics ESG score and its subscores are absolute measures, meaning that a ‘high risk’ assessment reflects a comparable degree of unmanaged ESG risk across all covered subindustries, (Sustainalytics, 2019). This implies that a financial company, for example, can be directly compared with a chemical company or any other type of company.

The sectoral scores, however, show that ESG ratings on average can be significantly different across industries. For example, scores are worse for Industrial Conglomerates, Steel companies and the Oil & Gas production companies suggesting that the manageability of risk is not independent from the absolute level of industrial exposure. In other words, economic value at risk from pollution is considered higher in the fossil fuel industries, and this risk cannot be fully offset by good risk management practices. In terms of aggregated ESG scores companies in the following industries rank the highest in terms of ESG performance in our sample: Textiles & Apparel industry, Transportation Infrastructure, Real Estate and Media. It should be remarked, however, that the sample size of some industries (Transport, Energy services, etc.) is rather small and this could obviously make it difficult to draw general conclusions of these sectors’ ESG performance. Also, this assessment does not cover the entire sector and based only on the listed companies in the sector, hence is not indicative for non-listed companies and for the given economic sector.

### 3.4 ESG rating and ranking of general stock indices

Table 3 presents the average sector specific ESG scores and ranks for general stock indices.

European indices rank higher in terms of arithmetic average ESG scores than indices in other continents. The MIB (Italy), DAX (Germany), OMXSTO (Sweden) are ranked on the top no matter whether the Sustainalytics or Refinitiv ESG scores are used. The RTS (Russia) and Hang Seng (Hong Kong, China) have the lowest ranking. The dispersion of ESG scores within the indices also increases as the average ESG score of the index decreases.

One should remark, however, that for many of the analyzed stock benchmarks, the presented ranking could be biased by the small sample size and that they show an arithmetic average and not weighted average. Results for Australia, Canada, Norway, Russia are especially exposed to the small sample size due to lack of data and to the impossibility to calculate weighted averages. The Budapest Stock Exchange’s stock index (BUX) is not shown, as there were only 2 companies (MOL and OTP) in the index for which Sustainalytics ratings were available.

**Table 3:** Sector specific ESG scores and ranks of stock exchange indices (sorted by Sustainalytics (SA) average rank).

Exchange	Country	SA		R		Rank	
		mean	stdev	mean	stdev	SA	R
MIB	Italy	87	8	77	19	1	6
IBEX	Spain	87	8	67	17	2	14
DAX	Germany	85	11	82	11	3	2
OMXSTO	Sweden	84	8	84	7	4	1
CAC40	France	84	11	71	16	5	9
AEX	Netherlands	82	8	75	14	6	8
SMI	Switzerland	81	13	79	15	7	3
OBX	Norway	77	12	76	14	8	7
FTSE100	United Kingdom	76	17	78	16	9	5
ATX	Austria	76	4	69	17	10	12
ASX	Australia	72	16	68	20	11	13
TSX	Canada	71	17	70	14	12	10
COPOMX	Denmark	71	10	78	12	13	4
SNP500	United States	70	16	63	19	14	18
BEL	Belgium	69	15	64	20	15	17
HELOMX	Finland	68	27	69	21	16	11
NIKKEI225	Japan	65	19	61	20	17	19
Hang Seng	Hong Kong (China)	64	16	64	22	18	16
RTS	Russia	63	23	66	20	19	15
**Average**		**71**	**17**	**66**	**19**		

**Notes:** In the table above, ‘SA’ denotes Sustainalytics and ‘R’ denotes Refinitiv. For ensuring comparability between Refinitiv and Sustainalytics scores, the Sustainalytics ESG absolute risk scores were transformed by changing the direction of the measurement scales and by calculating within industry group normalized scores for each company with the min–max normalization method.

To understand what factors drive ESG ratings one would need detailed information on the models of each rating provider and on input data of hundreds of indicators in the models. As this information is not publicly available due to confidentiality and business reasons, we analyze the issue by using publicly available company level data together with E, S, G scores and country level indicators. We regressed the ESG aggregated scores on the logarithm of book value, dividend yield (in %), on the country level sovereign credit default swap, (CDS, in basis points, bp) and the ESG disclosure rate (in %). The disclosure rate measures the proportion of an exchange’s large listings that disclosed the seven key sustainability performance indicators (employee turnover, energy, GHG emissions, injury rate, payroll, water, waste). In Eq. (5) *k* denotes the rating provider (either Sustainalytics or Refinitiv), i denotes the rated issuer, ESG is the Environmental Social and Governance score, α is the constant, *X’* the vector of explanatory variables β is the vector of explanatory variable coefficients and u is the error term.

(5) $$ESG_{i,k}=\alpha_{k}+X_{i,k}^{\prime }\beta_{k}+w_{1,k}\cdot E_{i,k}+w_{2,k}\cdot S_{i,k}++w_{3,k}\cdot G_{i,k}+u_{i,k}$$

In general, the model fit measured by the adjusted $R^{2}$s (0.84 in case Sustainalytics, 0.98 in case of Refinitive) are high, partly because the E, S and G scores were used as regressors (Table 4). An advantage of having E, S and G subscores in the estimated equation, beyond avoiding parameter biases from omitted variables, is that it allows testing the hypothesis of parameter equality for E, S and G subscores. The Wald test rejects the parameter equality hypothesis for all pairs of E, S and G coefficients at 1% significance level. The parameter of social subscore has the greatest value no matter whether Sustainalytics or Refinitiv data was used. Interestingly, the Sustainalytics risk subscores are equally weighted in the original methodology, but once they are converted into industry peer scores for comparability with Refinitiv, the equal weighting hypothesis cannot be held. Our estimated model does not identify factors which have significant impact on ratings of both rating providers and are not measured by subscores. The sign of country CDS is negative as expected (the higher the country level default risk, the lower the ESG score on average), although parameters are not significant. ESG disclosure rate coefficient is intuitively positive for both rating providers, although only significant in case of Sustainalytics. This discrepancy may be a result of differences in rating methodology, as transparency and company disclosure is at the core of Refinitiv methodology, (Refinitiv, 2022). Although not reporting ‘immaterial’ data points does not greatly affect a company’s Refinitiv score, not reporting on ‘highly material’ data points negatively affects a company’s score. The logarithm of book value has a positive estimated coefficient and significant value if the Sustainalytics data is used, suggesting that larger firms are either less exposed or expected to better manage ESG risks compared to peers in the industry. This may be related to economic factors not measured by the E, S and G subscores, still explaining variance in the ESG scores on the country level (see Table 4). .

**Table 4:** Regression results with Eq. (5).

	(1)	(2)
	SA_ESG	R_ESG

Book value (log)	0.158	0.0997*
Dividend yield (%)	32.54*	−2.819
Country CDS (bp)	−0.0210	−0.00477
ESG disclosure (%)	0.0827***	0.000396
SA_E	0.334***	
SA_S	0.494***	
SA_G	0.329***	
R_E		0.259***
R_S		0.427***
R_G		0.303***
Constant	−14.31***	0.777

$R^{2}$	0.84	0.98
$adj.R^{2}$	0.84	0.98
Root MSE	9.4	2.2
F (prob.)	0.00	0.00

Observations	839	650

* p<0.05, ** p<0.01, *** p<0.001.

**Notes:** In the table above, ‘SA’ denotes Sustainalytics and ‘R’ denotes Refinitiv. For ensuring comparability between Refinitiv and Sustainalytics scores, the Sustainalytics ESG absolute risk scores were transformed by changing the direction of the measurement scales and by calculating within industry group normalized scores for each company with the min–max normalization method.

### 3.5 Robustness analysis

Here, we quantify the uncertainty in the ESG score, which can demonstrate the extent to which issuer companies can be differentiated by their scores. Then, issuer company ESG scores are aggregated at the stock index level to compare stock exchange benchmarks in terms of ESG investment opportunities and their uncertainty.

There are many underlying assumptions of ESG ratings, which could be tested. Here, we examined three particularly important ones of these in our uncertainty analysis as discussed in Section 2.1 on the Methods. The assumptions were chosen as plausible alternative pathways in the construction of the ESG ratings in line with the literature on constructing composite indicators and ESG ratings: (i) the aggregation method [arithmetic or geometric mean or harmonic mean], (ii) the weights [Gaussian noise on the weights, ∑w = 1.], (iii) the data provider [Sustainalytics or Refinitiv].

**Fig. 2:**
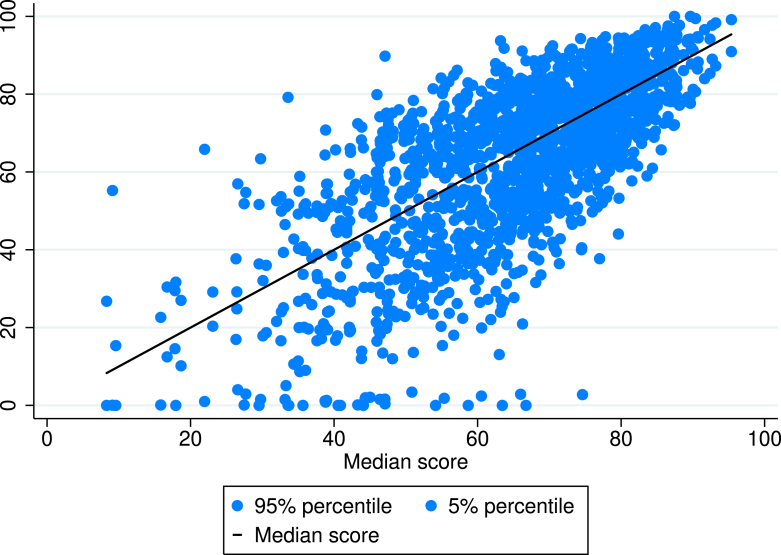
Monte Carlo simulation results — simulated ESG scores in the sample (median values (black dots) and 5% and 95% percentiles (light blue dots), ordered by median scores). **Notes:** Impact of varying randomly all three assumptions (1) Aggregation formula, (2) Weighting, (3) Data provider are taken into account.

To tackle the problem of zeros when using the geometric average formula, we replaced zeros by 0.01 values. As ESG subscores are expressed on the same scale, no further normalization was needed.

We performed a Monte Carlo experiment to test the above three assumptions, and re-built the ESG aggregated score 4000 times, each time with a randomly-selected combination of assumptions using the May 2022 observations of the ESG scores.

In general, the ESG aggregated scores are not very robust. ESG scores below the top decile are variant to methodological assumptions. Mid-scores can be stated to be within around ＋/−20 scores of precision, (Fig. 2). This finding could be used to guide the conclusions that can be based on the scores in general. For example, differences of 5–10 scores between mid-score issuer companies cannot be deemed as highly significant, whereas differences of 30 scores upwards or downwards can show a meaningful difference. The confidence intervals are generally narrower (＋/−5) for top-ranking stock issuers (above 90 scores), and wider for some low-ranking ones.

ESG investment strategies are often built upon specific strategies. For example, some investors apply (i) best-in-class or (ii) exclusion rules. Our simulation analysis reveals that only the best-in-class strategy can be effectively based on the ESG Scores of stocks in the analyzed stock indices. Our study does not aim to decide whether the score components correctly assess the expected outcomes of ratings. It can be stated, though, that best-in-class issuers can be differentiated from others, if one selects those stocks which are highly rated independently from data provider.

**Fig. 3:**
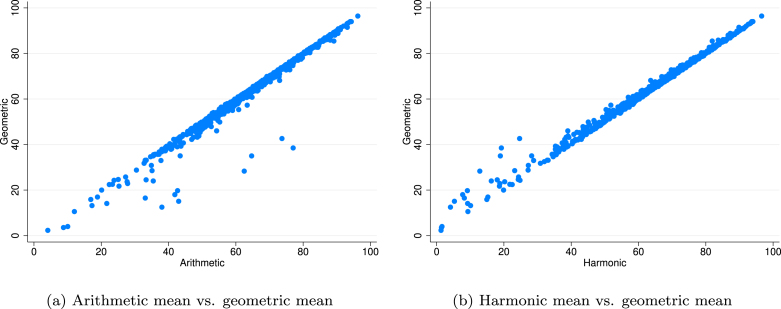
Monte Carlo results, simulated scores — aggregation rule (median scores).

It is also possible to compare the simulation results of the alternative assumptions for the aggregation rule. The choice of arithmetic, geometric or harmonic mean formula does not have meaningful impact on the ratings of the best rated stocks (Fig. 3). This finding could be important for those investors, who do not accept a trade-off approach of ESG components (for example, a substitution of good environmental performance by weak governance performance as discussed in Section 2.2).

**Fig. 4:**
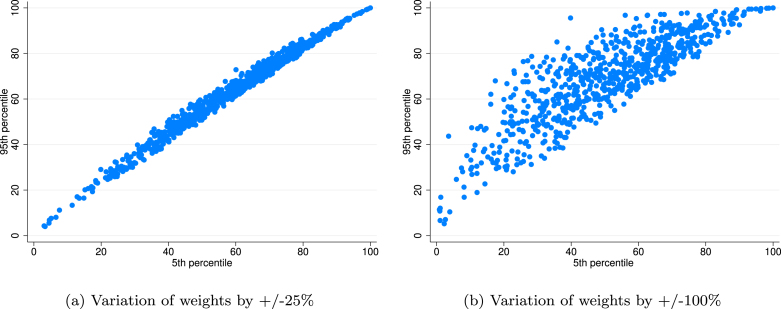
Monte Carlo results, simulated scores - weighting assumption (median scores).

The second assumption was the weighting of ESG ratings, which we tested as part of the Monte Carlo Simulations. We tested in separated simulations minor variation (＋/−25%) and major variation (+/100%) of weights. The latter implies that impact of zero/double weights of the original ($w_{i}$ =1/3) was tested. Fig. 4(b) shows that weights can have a meaningful impact only if major variation of weights is allowed (+/100%). In such case the mid-score variation range (between the 95th percentile ad 5th percentile) increases to ＋/−15 scores from about 7–8 scores in the baseline minor weight variation scenario (＋/−25%).

**Fig. 5:**
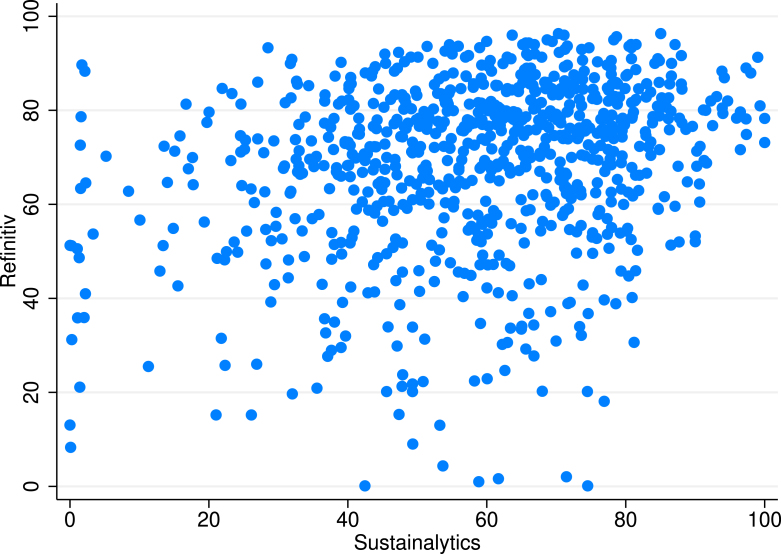
Monte Carlo simulation results – varying data provider (Sustainalytics vs Refinitiv) – median scores.

**Fig. 6:**
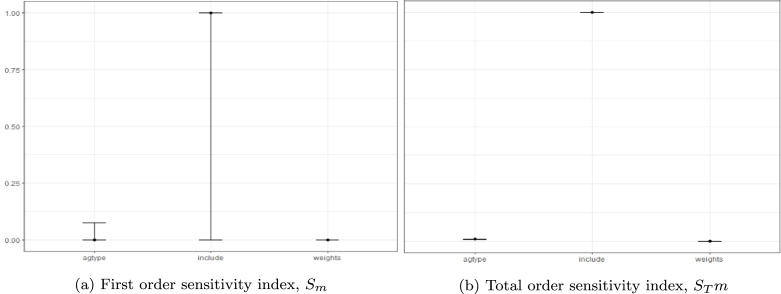
Variance-based sensitivity analysis results. **Notes:** Impact of varying randomly and simultaneously all three assumptions (1) Aggregation formula (*agtype*), (2) Weighting (*weight)*, (3) Data provider (*include*).

The third simulated assumption was the data provider. Fig. 5 reveals that the data provider choice has a major impact on the ESG evaluation of companies. Below 80 scores ESG assessment difference based on Sustainyalytics and Refinitiv becomes very wide (see Fig. 6).

The last step of the uncertainty assessment was the variance based sensitivity analysis of ESG scores as described in Section 2.1 on Methods. Both the  *first order sensitivity index* $S_{m}$ and $S_{Tm}$, the *total order sensitivity index* confirm that ESG assessment is most sensitive to the data source selection. This implies that the concept and measurement of ESG issues are not properly defined currently. Our results confirm the findings of Berg et al. (2019) that differences across rating providers drives the overall divergence.

**Fig. 7:**
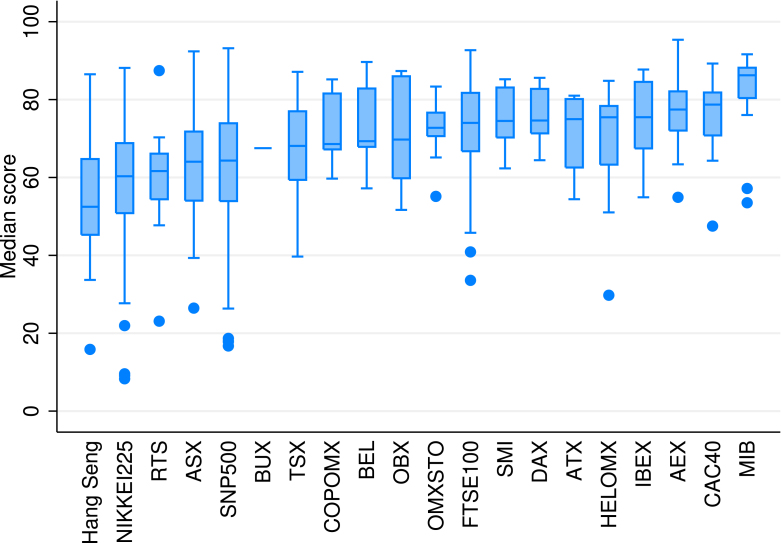
Box plot of ESG scores by exchanges, Monte Carlo simulation results (5%, 30%, 70%, 95% percentiles and median values). **Notes:** Outside values are dots, 95 and 5% percentiles are shown as upper/lower adjacent value whiskers. 70 and 30% percentiles are shown as upper/lower hinges, median are shown as mid values. Impact of varying randomly all three assumptions (1) Aggregation formula, (2) Weighting and (3) Data provider are taken into account.

Finally, the Monte Carlo simulation results are aggregated on the stock index level. Fig. 7 shows that European stock exchanges provide better ESG investment opportunities compared to other exchanges on average. The stock indices of Hong Kong–China (Hang Seng), Japan (Nikkei225) and Moscow (RTS) are on the other side of the distribution, perhaps probably due to the higher-share of industries with more controversies. It should be noted, that the uncertainty analysis changed significantly the position of some exchanges. For instance, the Helsinki Stock Exchange Index (HELOMX) is ranked relatively lower in the uncertainty analysis compared to the simple rankings based on ESG score averages. This finding confirms the challenges stemming from the substitution of E, S and G issues and is a reminder for investors to take the ESG scores always with a pinch of salt.

## 4 Policy relevance

The European Union’s Sustainable finance taxonomy - Regulation (EU) 2020/852 sets the ‘Do No Significant Harm’(DNSH) criteria. These criteria can be interpreted as a non-acceptance of perfect substitution of E, S and G issues, or at least the compensatory approach towards significant harms is not supported in the European Union. For the activities contributing to one or more of the six objectives qualifying as sustainable, the DNSH criteria requires that the activities cannot cause significant harm to any of the Taxonomy objectives. For every activity, the technical screening criteria (TSC) define compliance with do no significant harm by setting thresholds.

Stock benchmarks are also subject to the European Commission Delegated Regulation (EU) of 17.7.2020 requiring explanation on how ESG factors are reflected in stock benchmarks. In this study we discuss some of the metrics in the annex of the delegated act, including the average E, S, G scores. Our uncertainty analysis revealed significant divergence in ESG ratings of listed stocks, which may need to be addressed by policy makers and regulators. Administrators of EU Paris-aligned Benchmarks shall disclose in their benchmark methodology any additional exclusion criteria they use and which are based on climate-related or other environmental, social and governance (ESG) factors. They should display the corresponding score of the relevant ESG factors vis-à-vis the benchmark, at an aggregated value.

The Non-Financial Reporting Directive (NFRD) 2014/95/EU lays down the rules on disclosure of non-financial and diversity information by certain large companies. EU rules on non-financial reporting currently apply to large public-interest companies with more than 500 employees. This covers approximately 11 700 large companies and groups across the EU, including listed companies, banks, insurance companies, other companies designated by national authorities as public-interest entities. Required reporting is related to: environmental matters, social matters and treatment of employees, respect for human rights anti-corruption and bribery, diversity on company boards (in terms of age, gender, educational and professional background). On 21 April 2021, the Commission adopted a proposal for a Corporate Sustainability Reporting Directive (CSRD), which would amend the existing reporting requirements of the NFRD. The proposal extends the scope to all large companies and all companies listed on regulated markets and introduces more detailed reporting requirements, and a requirement to report according to mandatory EU sustainability reporting standards.

In the United States, the Securities and Exchange Commission (SEC) proposed to amend rules and forms to require registered investment advisers, registered investment companies, and business development companies, to provide additional information regarding their environmental, social, and governance (“ESG”) investment practices (SEC, 2022).

Exchanges have been playing a fundamental role in the development and stimulation of sustainable finance (Erhart, 2018). Exchanges have created a transparent green marketplace and served both issuer and investor sides of the market. Establishment of green listings was integral part of the Sustainable Stock Exchanges (SSE) Initiative announced in New York in 2009. The initiative has been a voluntary learning platform for encouraging sustainable investment organized by the UN involving partner exchanges, to provide sustainability-related indices and green listings.

## 5 Discussions

There are obvious limitations of our methodology and results, which should be clearly communicated. First, broadening the scope and sample of our analysis would help increasing punctuality of our results. For example, investigating further stocks and stock indices could be an obvious future research direction. Also, by using the ESG ratings from other rating providers could increase the robustness and precision of our results, as recommended by Berg et al. (2019). Exact weightings were not published for all the indices we covered, hence recalculation of our results by using market capitalization instead of equal weights could be a meaningful research option. Finding analytical and practical ways to deal with the insufficient correlation structure of ESG scores could also contribute to the development of ESG ratings and investments. Finally, one should not forget about the possible conflicts of interest embedded in ESG ratings, which may influence data quality and hence the reliability of our conclusions.

## 6 Conclusions

In this paper, we deal with new issues related to ESG ratings of stocks listed on 20 leading stock exchanges using data from two global ESG data providers: Sustainalytics and Refinitiv. Environmental, Social and Governance (ESG) ratings have been becoming an integral part of financial, business and consumption decisions. Phasing-in legal requirements in the European Union and in the United States are being imposed on a growing number of corporations and financial service providers to publish and integrate ESG information.

The key novelty of our study is that it extends the scope of the ESG discussion from ESG benchmarks to general stock benchmarks as these constitute the majority of the benchmark universe.

A common obstacle to the use of ESG ratings is that ratings of different rating agencies are often not directly comparable. The ratings of Sustainalytics and Refinitiv in our sample are not exemptions. The Sustainalytics ESG score is a risk score, while the Refinitiv score measures good performance. We transformed statistically the scores of Sustainyalytics onto the scale of Refinitiv, though there remained still substantial discrepancy in their ratings of the same stocks.

We show that listed stocks’ environmental, social and governance scores correlation ratio with the aggregated ESG scores is balanced and varies within the recommended 0.4–0.8 range for meaningful aggregates. However, the pairwise correlation between rating providers is weak. Also, correlation of environmental, social and governance scores for the same stock is low in case of Sustainalytics. All these may create a puzzle for service providers, regulators and investors in sustainable finance on how to reconcile and manage mutually environmental, social and governance risks.

We conduct a Monte Carlo simulation experiment recommended for composite indicators like ESG ratings to test the uncertainty in ratings, OECD-JRC (2008), Becker (2021). We examined three particularly important assumptions of ESG ratings: (i) the aggregation method [arithmetic or geometric mean or harmonic mean], (ii) the weights [Gaussian noise on the weights, ∑w = 1.], (iii) the data provider [Sustainalytics or Refinitiv].

In general, the ESG aggregated scores are not very robust, and users should take them with a pinch of salt. The choice of the ESG data provider has a major impact on the overall ESG evaluation of stocks, and large variation of weights has a minor impact. Below 80 scores ESG assessment difference based on Sustainyalytics and Refinitiv becomes very wide. Especially the lowest ESG scores are variant to methodological choices. This finding could be used to guide the conclusions that can be based on the ESG scores. For example, differences of 5–10 scores between issuer companies cannot be deemed as highly significant, whereas differences of 30 scores upwards or downwards can show a meaningful difference. ESG investment strategies are often built upon specific strategies. For example, some investors apply (i) best-in-class or (ii) exclusion rules. Our simulation analysis reveals that only the best-in-class rule can be effectively based on the ESG scores of stocks in the analyzed stock indices.

Finally, the Monte Carlo simulation results are aggregated on the stock index level. Aggregated results show that leading stock indices of EU exchanges provide good ESG investment opportunities compared to other international exchanges. General stock indices in Europe rank on the top of the 20 stock indices we investigated. Many stock indices in Europe belong to the best third of the ESG distribution, including benchmark stock indices in Italy, France and the Netherlands.

Sustainalytics, Refinitiv and many other data providers apply additive aggregation formula. Such a choice is common in the international practice of ratings and composite indicators, however, this methodological choice has important consequences. An undesirable feature of additive aggregations is the implied full compensation, such that poor performance in some indicators can be compensated for by sufficiently high values in other indicators. The correlation analysis showed that environmental, social and governance scores are not always highly correlated. Hence, on average one cannot find an investment portfolio building on the sample of stocks in the analyzed benchmark indices without trade-offs between environmental social and governance goals.

## CRediT authorship contribution statement

**Szilárd Erhart:** Conceptualization, Methodology, Data curation, Software, Writing – original draft.

## Declaration of Competing Interest

The authors declare that they have no known competing financial interests or personal relationships that could have appeared to influence the work reported in this paper.

## Figures and Tables

**Table A.5:** Sample summary statistics .

Index		**2020**				**2022**			
Name	N(i)	N(R)	%(R)	N(SA)	%(SA)	N(R)	%(R)	N(SA)	%(SA)
AEX	25	10	40%	17	68%	16	64%	16	64%
ASX	200	51	26%	51	26%	48	24%	48	24%
ATX	20	1	5%	6	30%	6	30%	6	30%
BEL	20	9	45%	10	50%	10	50%	10	50%
BUX	15	0	0%	2	13%	2	13%	2	13%
CAC40	40	7	18%	39	98%	35	88%	35	88%
COPOMX	25	9	36%	10	40%	10	40%	10	40%
DAX	30	2	7%	28	93%	27	90%	27	90%
FTSE100	100	60	60%	68	68%	63	63%	63	63%
Hang Seng	27	27	100%	27	100%	27	100%	27	100%
HELOMX	25	12	48%	12	48%	12	48%	12	48%
IBEX	35	17	49%	22	63%	21	60%	21	60%
MIB	30	11	37%	21	70%	20	67%	20	67%
NIKKEI225	225	139	62%	140	62%	134	60%	137	61%
OBX	25	8	32%	9	36%	8	32%	8	32%
OMXSTO	30	7	23%	23	77%	23	77%	23	77%
RTS	50	0	0%	18	36%	17	34%	18	36%
SMI	20	0	0%	19	95%	18	90%	18	90%
SNP500	500	324	65%	465	93%	444	89%	444	89%
TSX	224	28	13%	29	13%	29	13%	29	13%

Average	1666	722	43%	1016	61%	970	58%	974	58%

**Notes:** In the table above, ‘SA’ denotes Sustainalytics, ‘R’ denotes Refinitiv. Percent values show the ratio of index component stocks in the sample compared to the total number of stocks in the given index shown the second column of the table N(i).

**Table A.6:** Sustainalytics ESG scores by sectors, 2020 (sorted by ESG score).

Industry	E	S	G	ESG	No of obs	Rank
Textiles & Appa	1.2	6.0	6.2	13.4	17	1
Transportation	1.7	7.4	4.9	14.0	4	2
Real estate	3.7	4.9	6.0	14.7	55	3
Media	0.4	7.0	7.5	14.9	22	4
Retailing	2.7	7.5	5.3	15.5	29	5
Technology hard	2.9	6.6	7.1	16.6	30	6
Containers & Pa	11.4	1.7	3.8	17.0	6	7
Consumer durabl	5.2	7.0	5.3	17.5	9	8
Auto components	3.9	6.3	7.6	17.8	9	9
Software & Serv	0.8	10.3	6.9	18.0	55	10
Building produc	9.0	2.6	6.5	18.1	11	11
Paper & Forestr	10.6	3.0	4.6	18.2	6	12
Commercial serv	2.9	10.3	5.8	19.1	25	13
Homebuilders	6.5	7.5	5.4	19.4	6	14
Telecommunicati	2.9	9.8	7.2	19.9	26	15
(...)						
Food products	8.7	11.2	6.3	26.3	45	27
Pharmaceuticals	1.3	15.3	9.8	26.4	53	28
Machinery	6.8	12.8	7.7	27.4	34	29
Chemicals	13.0	6.9	7.5	27.5	37	30
Refiners & Pipe	14.4	8.6	4.6	27.6	13	31
Automobiles	7.7	10.1	9.9	27.7	18	32
Energy services	10.0	9.2	8.9	28.0	4	33
Construction Ma	16.3	4.4	7.6	28.2	7	34
Utilities	13.5	10.2	6.4	30.1	58	35
Construction &	6.3	14.3	11.7	32.3	12	36
Diversified met	14.8	10.7	7.0	32.5	13	37
Aerospace & Def	8.5	16.5	7.8	32.7	11	38
Steel	16.1	12.7	7.6	36.4	12	39
Oil & Gas produ	18.3	10.0	8.9	37.1	38	40
Industrial cong	13.5	14.4	11.2	39.0	8	41
**Grand total**	**6.0**	**9.7**	**7.8**	**23.6**	**1016**	

**Notes:** The table shows the arithmetic average of Sustainalytics original ESG risk scores across industries. The data was collected in November 2020 from Yahoo Finance.

## Appendix Sample summary

See Table A.5, Table A.6.
